# The μSCAPE System: 3-Dimensional Profiling of Microfluidic Architectural Features Using a Flatbed Scanner

**DOI:** 10.1038/srep22246

**Published:** 2016-02-29

**Authors:** Kerui Xu, Qian Liu, Kimberly R. Jackson, James P. Landers

**Affiliations:** 1 Departments of Chemistry, University of Virginia, Charlottesville, VA 22904, USA; 2 Departments of Mechanical and Aerospace Engineering, University of Virginia, Charlottesville, VA 22904, USA; 3 Departments of Pathology, University of Virginia, Charlottesville, VA 22904, USA

## Abstract

We developed a microfluidic scanner-based profile exploration system, μSCAPE, capable of generating high resolution 3D profiles of microstructure architecture in a variety of transparent substrates. The profile is obtained by scanning the dye-filled microstructure followed by absorbance calculation and the reconstruction of the optical length at each point. The power of the method was demonstrated in (1) the inspection of the variation of the cross-section of laser-ablated PDMS channel; (2) the volume of PeT chamber; and (3) the population distribution of the volumes of the micro wells in HF-etched glass and laser-ablated PDMS. The reported methods features low equipment-cost, convenient operation and large field of view (FOV), and has revealed unreported quality parameters of the tested microstructures.

The concept, methods, capabilities and technologies associated with a ‘lab-on-a-chip’ have evolved dramatically since 2001 when a journal under the very name was launched^1^. From a single channel, single-function microchip to highly integrated systems^2^, and silicon- or glass-based microstructures to disposable polymer or paper based chip^3^, ‘lab-on-a-chip’ systems have begun to tackle real-world analytical problems in a diverse spectrum of fields ranging from pathogen detection^4^ to clinical diagnosis^5^,^6^, and even into forensic genotyping^7^,^8^. As the technologies of the lab-on-a-chip embrace the outside world with more and more devices productized and commercialized^9^,^10^ the quality control of the microdevice feature integrity become a significant issue that is critical to the success of the product.

In order to examine the geometric quality of the micro-devices the 3D profile of the target structures needs to be obtained. Commonly used profilometry techniques include stylus profilometry^11^, atomic force microscopy (AFM)^12^, confocal scanning microscopy (CSM)^13^ and scanning white light Interferometry (SWLI)^14^. All of these techniques have their individual pros and cons in terms of data acquisition rate, vertical/lateral resolution, and suitability for different applications^15^. However, due to the sophistication of microscale profilometry, these methods require delicate, sophisticated, high-cost instrumentation and well-trained personnel, even though manufacturing costs may have been reduced through mass production. The capitalization of labor and instrumentation for quality control may come with a significant financial burden, one beyond the resource capabilities of start-up companies and like innovators. Furthermore, the limited FOV that can be probed by these methods (normally on the order of tens of mm^2^) make them less desirable for the inspection of the microdevices with relative large areas and/or consisting of multiple domains. This is exacerbated when quality control inspection is required during mass production.

In this report, we describe our findings on the use of an office flatbed scanner to detect the 3D profiles of microfluidic structures. In 2010, we reported that, by measuring the absorbance of dye solution after it passed through the microchannels, the quality of the channels could be easily determined spectroscopically^16^. Here, we exploit absorbance of dye-filled channels again, but in a very different way. The fundamental principle here is the measurement of the absorbance of a dye solution while it is still in the microfluidic architecture. This process can be summarized in three steps: (1) filling of the microstructures with the colorimetric dye solution that has characteristic absorbing wavelength. In this work, we exploited Allura Red solution; (2) scanning for image acquisition of the microfeatures at the 2-μm level; and (3) for each pixel of the microstructure image, the depth of the structure is calculated based on Lambert-Beer’s law using the absorbance of that pixel and the pre-determined extinction coefficient of the dye solution. Broadwell *et al.*^17^ reported using the absorbance of a dye to optically-represent the dimensions of a microfluidic structure imaged by a bright field microscope-CCD (charge-coupled device) system. The accuracy of the absorbance-based 3D-profilometry has been proven comparable or even better than the typical stylus-based profilometry. However, the limited number of pixels in the square CCD (256 by 256) compromises the FOV of this method restricting it to several square millimeters. For the detection of larger areas, moving of the sample and stitching of the images are required. Although microscope CCD cameras with higher resolution (e.g., 1024 by 1024 or even higher number of pixels) are available in recent years, the price for larger CCD increases disproportionately versus sizes. Alternatively, by using a completely different strategy for imaging, a flatbed scanner can achieve a much larger FOV with a low-cost, built-in line CCD. By moving the line CCD across the entire scanning field, a large, high-resolution detection window can be interrogated (at the tens of gigapixels level); which is technically (and economically) unrealistic to achieve using a single, static square CCD chip. The area of the FOV presented by a scanner is on the order of hundreds of cm^2^, with a resolution down at the 2 μm level^18^. This allows the scanner to interrogate, simultaneously and in parallel, a large number of bioassay well-plates^18^.

In addition, during the scanning process the color in each scanned image is split into red, green and blue (RGB) space by a pre-filtering process prior to being detected by the CCD^18^. Exploiting the values in RGB space has enabled scanner-based detection with various types of colorimetric assays including inorganic ions^19^,^20^, organic compounds^21^ and bacterial identification^22^. The RGB space also allows monochromatic absorbance measurements to be acquired without external optical filters that otherwise would be required in most of the monochromic microscope CCDs. Measurement of absorbance or optical density (OD) in a single RGB channel enables quantitative analysis in a number of different formats, and has been applied to bacterial susceptibility testing^23^, diffusion-based dynamics of dye solution^24^ and scanner-based quantitative detection of polyphenol^25^. In this paper, we demonstrate the power of a conventional office scanner to examine the qualities of microfluidic structures including: (1) laser-ablated PDMS channels, (2) polyester-toner (PeT) film-derived chambers and, (3) micro-well arrays fabricated either in glass by HF or in PDMS by laser-ablation. The capabilities (and limitations) of a flatbed scanner for evaluating the geometric parameters of microscale features and quantitatively defining their uniformity is demonstrated.

## Material and Methods

All reagents, unless specifically indicated, are all purchased from Sigma-Aldrich (Oakville, ON).

### Preparation of the dye solution

10X TE(pH 7.5)-glycerol mixture was used as the solvent for the dye solution to minimize evaporation and the difference in refractive indices between the dye solution and glass/PDMS (Supplementary Information, Section 2).

Allura red solution was prepared by dissolving in 10X TE buffer-glycerol mixed solvent at desired concentrations (specified in following sections) as stock solutions for future use.

### Fabrication of the microstructures

Standard channels for the determination of the extinction coefficient of the dye solution were fabricated by conventional photolithography and HF etching in glass. The mask was designed using AutoCAD and then printed in photographic film in high resolution (Thin metal parts, CO). Photoresist-chrome layers coated borofloat glass plate (Telic, Valencia, CA) was etched by HF solution (HF/HNO_3_:200/30 (v/v)) after standard procedures of photolithography. The remaining photoresist and chromium on the plate were removed completely after the etching. The glass channels were dried by nitrogen purging and the depths were determined by stylus profilometry(KLA-Tencor, Milpitas, CA).

PDMS channels are fabricated by laser ablation into cured PDMS plate. Briefly, PDMS plates were prepared by mixing of monomer and curing reagent at mass ratio of 10:1 followed by oven heating at 70 °C for two hours. Channel are designed in CorelDRAW 10.0 (Corel Corporation, Ottawa, Canada), and then ablated by VersaLaser VLS 3.50 with 50W CO_2_ laser source (Universal Laser System Inc.) into PDMS plate. Power settings of 30%, 60% and 90% were used to make channels with different depths. The fabricated PDMS channels were cleaned by methanol wash and dried by nitrogen purge. Access holes are punched at both end to be the inlet and outlet of the channel.

Round PeT chambers are fabricated following the previously described procedures^26^ with designed volume of 2.5, 5, 10, 20, 30, 40 and 50 μL. Briefly, polyester films ((CG5000, 3M) were printed with black toner (HP C-4127X) and then cut by laser cutter to form the body of the chamber, followed by lamination at 130 °C with polyester film to form an enclosed chamber.

In the fabrication of the chambers on the centrifugal microfluidic devices for DNA extraction, to eliminate the sagging of the chamber, the assembled microdevice was sandwiched between pre-cut pieces of ~1 mm thick brass shimstock before lamination.

Micro well arrays are fabricated in two different ways:

- (1) HF etching in glass using the methods described above. Designed diameter of the well was 80 μm and the etched three different depths were achieved at about 7, 10 and 14 μm, measured by stylus profilometry.
- (2) Laser single-pulse ablation in PDMS. Briefly, the power density of the laser cutter was set to 200 points per inch (PPI) and parallel lines with spacing of 150 μm were ablated into the cured PDMS plate at speed 100% and three power settings were used at 0.5%, 1% and 1.5%.

### Scanning settings and image analysis

Images were scanned by EpsonV600 in sRGB space and the scan mode was selected to “film scan” instead of commonly used “reflective” mode to avoid shadow and reflection problem (Supplementary Information, section 1). “Film scan” mode is a common function in most of the flatbed scanners and allows for scanning of transparent medium such as films.

The scanned images were saved in TIFF format with 16 bit RGB color space. The saved images were imported into ImageJ and split into three color spaces (Red, Greed and Blue) for the subsequent calculation of absorbance.

The absorbance of the dye-filled microstructures are determined using A = log(I_0_/I_t_), in which I_0_ and I_t_ are both in the unit of 16 bit grayscale (between 0 to 65535) and correspond to the intensity of the incident light and the transmitted light, respectively. The calculation of each point’s absorbance and profile plotting was conducted in Matlab R2013b (MathsWorks, Natick, MA).

### Determination of extinction coefficient

The extinction coefficient of the dye solution was determined by measuring the absorbance of (1) different thickness of dye solutions at fixed concentration, or (2) different concentration of dye solutions at fixed thickness.

To obtain absorbance-depth standard curve, glass standard chambers with different depths (7.9, 15.7, 26.9, 49.4, 89 and 160 μm) were filled with 6 mM allura red solution, followed by scanning and image analysis in blue spaces.

To obtain absorbance-concentration standard curve, 27 μm glass standard chambers were filled with different concentrations of allura red solution (1.5, 3, 6, 12, 24 and 48 mM), followed by scanning and image analysis in blue spaces.

Blank solvent was used to fill the channel as the reference for the calculation of absorbance.

### Examination of the cross section of laser-ablated PDMS channels

The laser ablated PDMS channels were reversibly bonded onto a glass plate (Corning, NY) and then filled with 6 mM Allura solution and blank solvent as reference. The filled channels were scanned at 12800 dpi and the depth of channel at each pixel was calculated using the absorbance of that pixel and the extinction coefficient determined in section 2.4. The cross-sectional profile of the channel is reconstructed, then compared with the true profile of the channel examined by cut-and-check through microscopy.

### Examination of the volume of the PeT chambers

The PeT chambers were filled with 1.5 mM Allura red solution. Multiple filled chambers in a single device were scanned together at one time followed by image analysis, using blank solvent as the reference. The resolution of the scanning is 1200 dpi. The volume of each chamber was calculated by adding up all depths of the pixels of the chamber image and then multiplied by the area of a single pixel.

### Examination of the volume of the micro well array

The micro well array in glass was filled with 48 mM Allura red solution and pressed against a PDMS plate to be sealed, and the excessive dye solution was absorbed away by Kimwipe tissue. The micro well array in PDMS was filled with 48 mM Allura red solution and pressed against a glass plate to be sealed, and the excessive dye solution was absorbed away by Kimwipe tissue.

The filled micro wells were scanned at 12800 dpi (2 μm per pixel) and analyzed using blank solvent as the reference. The volumes of all the micro wells in the array were calculated and the distribution of the volume of the examined array is obtained

## Results and Discussion

### Determining the extinction coefficient

**Figure 1:**
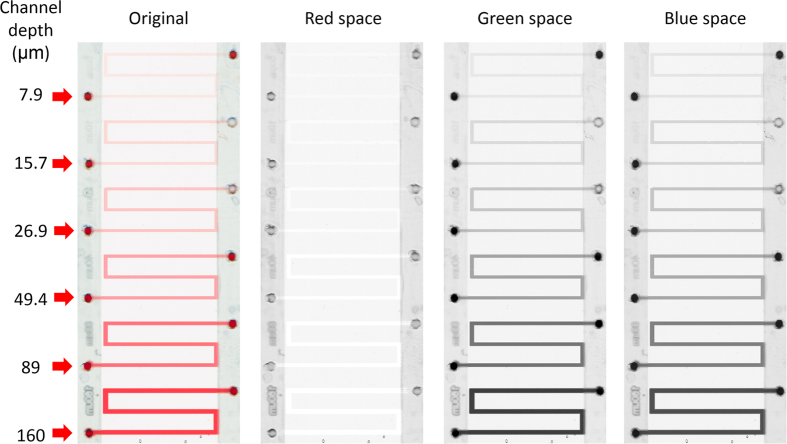
The scanned image of the standard glass channels filled with Allura Red solution (6 mM in 10XTE-glycerol mixed solvent). From left to right: the original image, the split images in red, green and blue space.

The scanned images of the channels filled with Allura Red dye solution shows different values in each of the RGB spaces (Fig. 1). The red space is almost identical to the white background, indicating that this dye absorbs little or no light in the wavelength red region. However, in the blue and green space, the fluidic channel shows an increasing grayscale as the depth increases. Blue space was selected for the absorbance test because of the greater extinction coefficient appeared in this channel reflected by darker images and better linearity.

**Figure 2:**
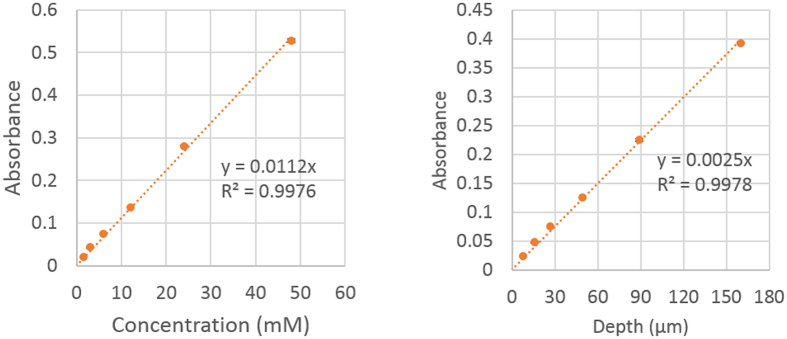
The calibration curve of absorbance vs. concentration (left) and depth (right) of alura red solution in the blue space (n = 3).

In order to create calibration curves, absorbance of either a fixed concentration of dye in standard channels (with known depths), or a series of standard solutions (at known concentration) in a channel with fixed depth, are measured. When the data are plotted in blue space, both channel depth and dye concentration have a linear relationship with absorbance of the Allura Red (Fig. 2). This proportionality of the absorbance to both the dye concentration and the optical length verified the applicability of Lambert-Beer’s law in the scanner-based absorbance measurement, and the subsequent extinction coefficient for Allura Red was calculated to be 0.00052 mM^−1^ μm^−1^. As a result, the Allura Red dye concentration could be selected based on the estimated structure depth, and determined extinction coefficient in Fig. 2 to assure the proper absorbance is below 0.6.

After establishing the appropriate calibration curve, the feature depth at any particular micro-locale in a given architecture can be back-calculated based on the absorbance value at that point using the concentration of the dye used and the associated extinction coefficient. With a lateral resolution at the 2-μm level, a **M**icrofluidic **SCA**nner-based **P**rofile **E**xamination (μSCAPE) system results for easily interrogating channels as small as ~100 μm in diameter and 10 μm in depth.

### Cross-sectional examination of PDMS channels

To test the capability of the μSCAPE system for defining 3D architecture, we evaluated microchannels that had been laser-ablated in PDMS. PDMS is the most commonly used polymer in the microfluidic community, and microstructures can be fabricated into this material typically through ‘pour and cure’ molding, but also via laser ablation. Molded PDMS microstructures present fine and uniform features but require multiple steps for masking and lithography, and require generation of a new master when changing the design. Laser ablation, on the other hand, is a simple, faster process, requires no masking, but creates more irregular features in comparison with molding^27^. Examination of the laser-ablated PDMS structures is difficult to achieve with stylus profilometry (due to the elasticity of the polymer), and the large slope from the channel edge to the center of the cone-shaped channel can lead to ambiguities when using scanning white light interferometry^28^. Moreover, the commonly used ‘cut-and-check’ method^27^ is limited as it can only interrogate the cross-section at the cutting plane, leaving the analysis devoid of a thorough, 3D view of the structure(s). Finally, the ‘cut-and-check’ method causes irreversible damages to the PDMS microstructures and voids the possibility of future use, which is unnecessarily wasteful in an era where green chemistry is gaining increased attention.

**Figure 3:**
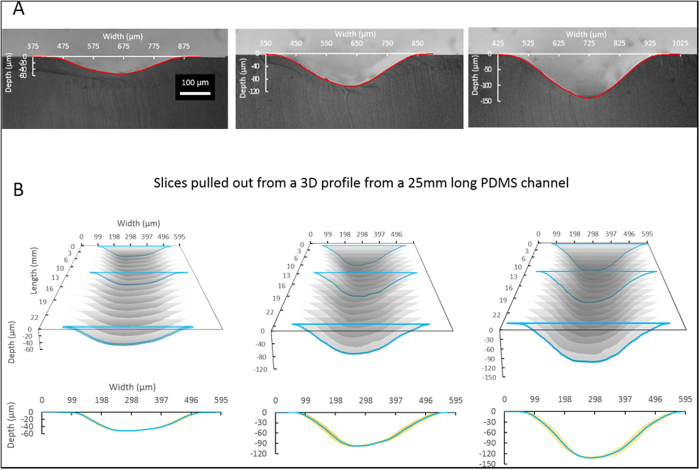
The examination of the cross-section of laser-ablated PDMS channels. (**A**) The comparison of the scanner-acquired profile (red) and the actual profile revealed by microscopy. (**B**) The inspection of the longitudinal cross-section variation of 25 mm PDMS channels ablated at different power setting. The interval of ±1 standard deviation of the 16 slices of cross-sectional profiles are shown in yellow shade.

Channels created by laser ablation at different power settings were evaluated with the μSCAPE system and compared with the actual profile examined under bright-field microscopy (Fig. 3A). It is obvious that the cross-sectional profiles obtained with μSCAPE (red line) are in good agreement with the bright field micrographs. This validated the feasibility and accuracy of the μSCAPE profiling in two dimensions. However, as a result of the large detection area of the scanner, we were also able to check the variation of the cross-sectional profile over a longitudinal distance of the channel (2.5 cm). Scanning three different 2.5 cm long PDMS channels, each ablated at different laser power settings, we were able to assemble a 3D profile for each from a series of 12,500 cross-sectional slices. Figure 3B shows a set of 16 of these slices evenly-spaced over the length of that channel to compose a longitudinal 3D profile. Despite that fact that this is a low resolution image (16 of 12,500 slices), differences in the profiles are clear, specifically: (1) deeper channels have more dimensional variation than shallower channel, which is due to the differences in the applied laser power, and (2) there is more variation in the side wall of the channel than there is in the floor. This may be explained by the mode of ablation (a single stroke of the laser beam), where the higher side wall variation may be attributed to the power fluctuation in the dispersion region of the Gaussian-shaped focal spot of the laser. It is clear that the larger the number of slides used to re-construct the channel dimensions, the higher in resolution the image will be. After the PDMS channels were scanned, they were rinsed with ethanol and no solvent-induced swelling or dye-induced fouling was observed.

In comparison with direct microscopic interrogation, the scanner-based profiling is more favorable in two ways: (1) the method is completely non-invasive and the microdevice remains functional for use after examination, and (2) one can quickly check the cross-sections of the microstructure at any point in any orientations after the image is acquired. It is noteworthy that the μSCAPE approach can interrogate relatively long channel distances, as this this limit is set by the scanner ‘scanning window’ which, in our case, is 250 mm by 8 mm. For proof of principle here, we chose three 25 mm long channels scanned simultaneously, as this is more relevant to the feature scale of common microfluidic devices.

### Examination of the volume of the PeT chamber

We have recently reported on a new fabrication method for multilayer microdevices using laser printed, cut and laminated (PCL) polyethylene terephthalate with toner-based bonding^29^. These polyethylene-toner (PeT) devices represent an emerging low-cost substrate for fast and convenient fabrication using fairly common office or lab equipment; a number of applications have been addressed with PeT microdevices, including microchip electrophoresis^30^,^31^, DNA extraction^32^ and centrifugal microfluidics^26^. However, the volume of the PeT chamber may be subject to variation due to the expansion of the polyester film during the heat lamination step; this is of particular concern with larger chambers (>5 mm diameter) that have the potential to deform due a ‘sagging’ effect in the capping layer, which ultimately affects accuracy in achieving a desired volume. While the theoretical chamber volume can be defined by stylus profilometry or SWLI prior to lamination of the capping layer, there are two obvious limits to this approach. First, these methods cannot be used to probe a closed chamber. Thus, they provide no information about the chamber after it has been capped and laminated, and no empirical volume measurement can be easily made to assure that no feature deformation has occurred. Second, larger microdevices (e.g., CD-sized centrifugal microfluidic devices) are more challenging for stylus profilometry or SWLI, as the area that can be probed by these methods is limited. It is in this respect that the μSCAPE approach addresses a significant need.

**Figure 4:**
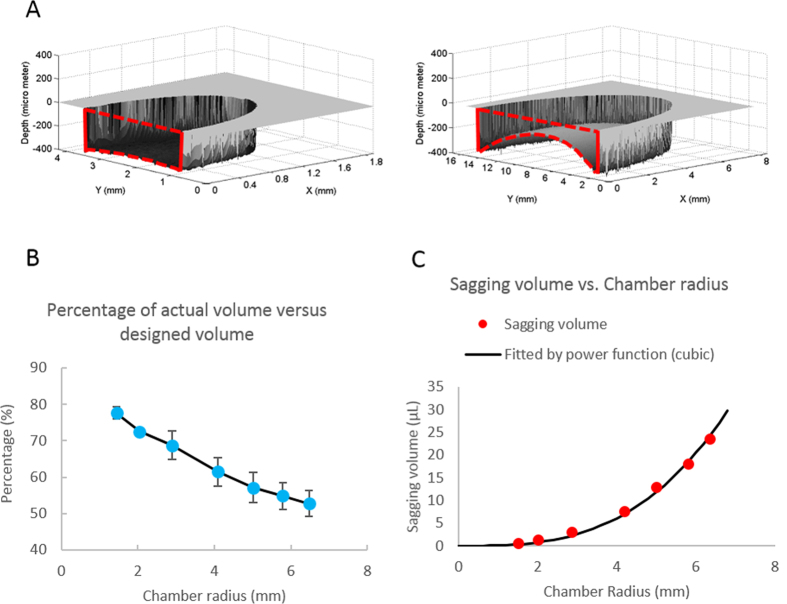
The volume determination of PeT chambers. (**A**) the cap deformation of a d = 2.5 mm PeT chamber (left) and a d = 13 mm PeT chamber. The curve part of the red dash line in the cross-sectional cut shows the sum of the deformation of both the floor and the ceiling of the chamber. (**B**) The actual volume as the percentage of the designed volume (n = 3). (**C**) The decreased volume of the chamber versus the radius of the chamber. (n = 3).

The 3D profile in Fig. 4A shows the visualization of a deformed chamber surface using the μSCAPE approach. Inherent in the optical length (absorbance) reconstruction is the assumption that the opposing surface is flat. This is certainly the case when profiling the glass or PDMS channels described in the previous section, where at least one surface of the channel is, indeed, flat. However, in the case of a post-laminated PeT chamber, there is potential for both the capping (ceiling) and base (floor) layers to be deformed. The reconstructed model shown in Fig. 4A is actually the sum of the deformation for both the upper and lower surfaces. While this may not accurately reflect the ‘true’ inner surface profile of the chamber, the volume determined for the chamber can be accurately calculated.

The μSCAPE-calculated volumes are given as the percentage of the theoretical volume expected based on the feature design. Figure 4B clearly shows that, not surprisingly, as the radius of the chamber increases, the deformation (sagging) of the chamber cap becomes more severe; for the largest chamber in this series, the actual volume is only ~50% of the volume predicted by design. This trend with volume deviation vs. chamber size is anticipated since the designed volume is proportional to the square of radius (r^2^) but the decreased volume (what we term the sagging volume) is proportional to the cubic of radius (r^3^) (Fig. 4C and Supplementary Information section 3).

**Figure 5:**
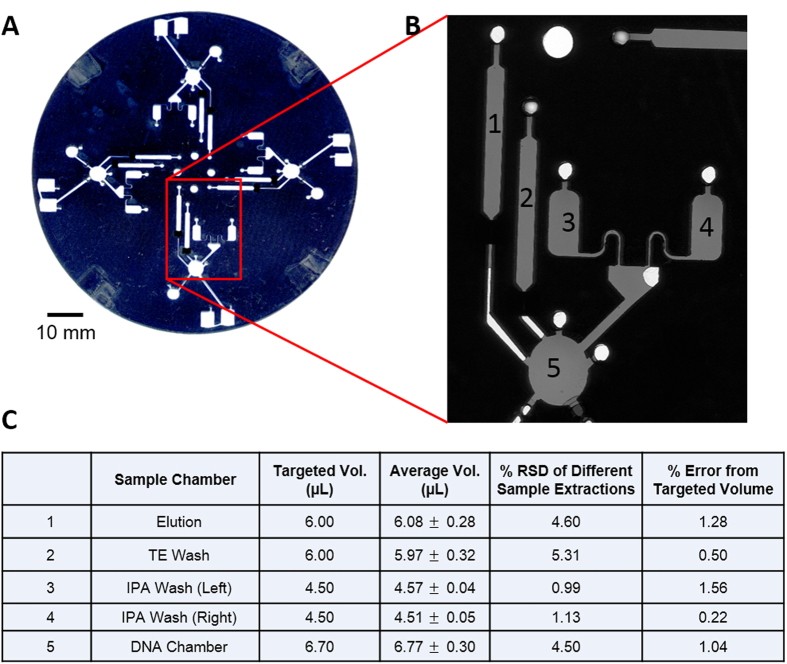
Volume determination of the PeT centrifugal microfluidic device for DNA extraction. (**A**) The scanned image of the full PeT device (unfilled). There are 4 identical domains symmetrical to the center of the disk. (**B**) The scanned image of a single domain containing 5 different chambers filled by Allura red solution in Blue space. (**C**) the results of the volume determination.

To correct this volume deviation problem in the PeT chamber, we developed an improved fabrication protocol where, during the lamination and bonding steps, the pre-assembled microdevice was sandwiched between pre-cut pieces of ~1 mm thick brass shimstock for lamination. An integrated PeT microfluidic device for DNA extraction was fabricated using the improved protocol, and the volumes determination are shown in Fig. 5. The μSCAPE method revealed that the improved protocol had less than 2% error from the targeted volume in the design with ~5% volume variance.

### The 3D profiling of array of micro-wells

One of the most appealing features of analytical microfluidic systems is the potential to exploit small features for higher throughput bioassays, and subsequently generate large amounts of data. The integration of thousands or even millions of assays on a single microdevice has been realized by micro well arrays, and a number of diverse applications have been reported including DNA quantitation^5^,^33^,^34^ and single cell genotyping^35^.

To obtain accurate and reproducible results from micro-well arrays, it is essential to assure that certain critical geometric parameters, like micro-well volumes, are constant; e.g., in the determination of the DNA concentration by digital PCR^5^,^33^,^35^. Previous studies using arrays with a large number of micro-wells usually assume uniform well volumes in the processing of data, primarily because of the unavailability of statistical information on the micro-wells. In large arrays, it is important to define the well-to-well variation in volume, as well as identifying ‘bad’ wells whose volume exceeds some specifications (i.e., standard deviation) that have been determined to be acceptable a priori. However, if only inspected by random low-number sampling of select micro-wells in the large micro-well array, the probability of identifying the ‘bad’ wells is low. This can be solved by a comprehensive inspection of the entire array. We demonstrate that the μSCAPE approach allows this to be carried out with micro wells fabricated by two different methods; conventional HF etching in glass and laser ablation in PDMS. Moreover, the scanner-based profiling effectively identifies wells in an array that fall outside the accepted volume standard deviation.

### Comparison of different fabrication methods

**Figure 6:**
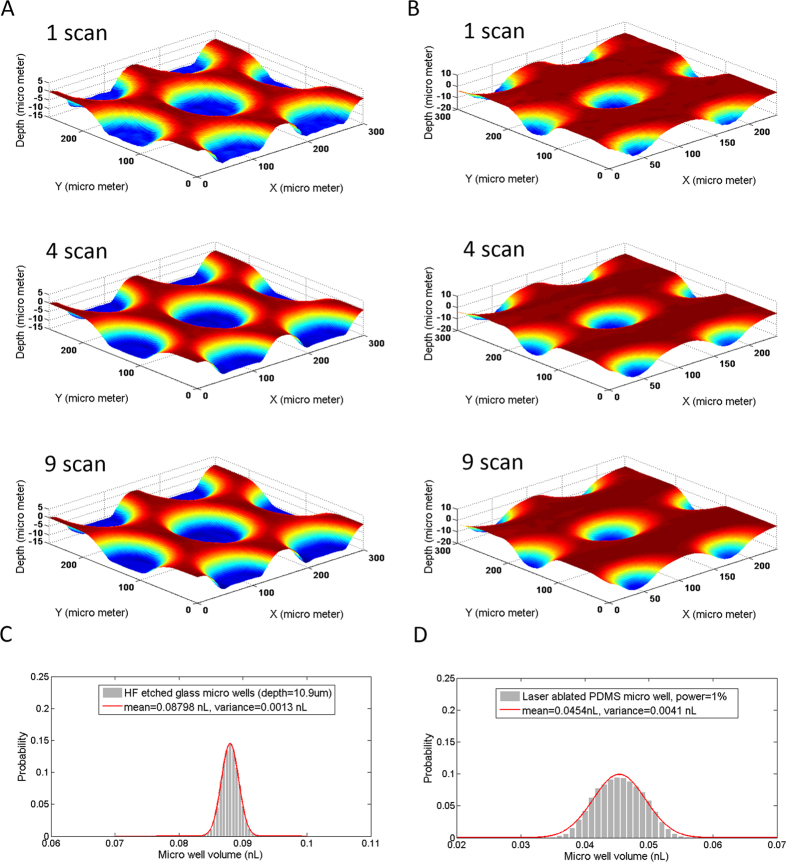
The volume distribution of micro wells in HF-etched glass and laser-ablated PDMS. Samples of 3D profiles of micro wells in glass (**A**) and PDMS (**B**) obtained in single scan, 4 scans and 9 scans, from top to the bottom. The volume distribution of the glass and PDMS micro wells is shown in (**C**,**D**), respectively.

The 3D scanner-based profiling of micro-wells involves features that are significantly smaller than those described in the previous sections. As a result, the resolution needs to be much higher than 1200 dpi used earlier in the scanning of the PeT chambers and, specifically in this case, 12800 dpi. Associated with the higher resolution is an increased noise level, which may mask certain features. In order to improve the S/N ratio to more effectively reveal the profiles of the micro-wells, multiple scans were carried out followed by an averaging of the profile. Figure 6A,B show the 3D profiles of micro-wells in HF-etched glass and laser-ablated PDMS, with averaged images resulting from 1, 4 and 9 scans. It is noteworthy that the surface noise (roughness) of the well profile in a single scan is too large to correctly reveal the details of the structure, and that multiple 3D scans of each micro-well array were needed to average out the noise. Theoretically, the average of N scans will give a reduce the noise by a factor of 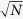. It is obvious that the 4-scan-average is much smoother than the single-scan profile, and the 9-scan-average enhances it further, but without significant improvement relative to 4-scan average. Therefore, to adequately balance the ‘scanning time’ with the resultant ‘profile quality’, 4 scans were used for analysis. Due to the differences in these fabrication modes, the shape of the HF-etched glass micro-wells appears to be a basin-shaped structure with a flat bottom, while laser-ablated PDMS micro-wells shows a cone-shaped pit.

The volumes of 10,000 wells were calculated from the average of 4 3D scan profiles of the glass and PDMS micro-well arrays (Fig. 6C,D). For glass micro-wells, the defined volumes show a Gaussian distribution with a mean of μ = 0.0880 nL and variance of σ = 0.0013 nL, while the laser-ablated PDMS micro-wells have a mean of μ = 0.0454 nL and a variance of σ = 0.0041 nL. The apparent reduction in quality of the laser-ablated PDMS micro-wells might be attributed to the variation of focal distance across the substrate, and the variation of the output power of the laser pulse.

To investigate the quality of these two methods, we fabricated micro-wells under different conditions. For HF-etched glass, we created micro-wells with three different depths, facilitated by increasing the etch time of (with same designed diameter of 80 μm), and for laser-ablated PDMS, micro-wells with three different depths were created by increasing the power output of the laser pulse. Both methods show increased variance of the volume as the well depth is increased, but the coefficient of variance (percentage of variance/mean) decreases (Tables 1 and 2, and Figure S3 in Supplementary Information). In addition, the mean of the μSCAPE-defined volumes associated with the HF-etched glass wells compared favorably with the theoretical volumes (Figures S4 and S5).

Overall, the information revealed represents the first comprehensive whole-population-scale quality assessment of the volume distribution of the micro wells arrays, which also provides a useful guidance for the selection of fabrication conditions in order to control the quality of manufacturing.

### Inspection of multiple well types

**Figure 7:**
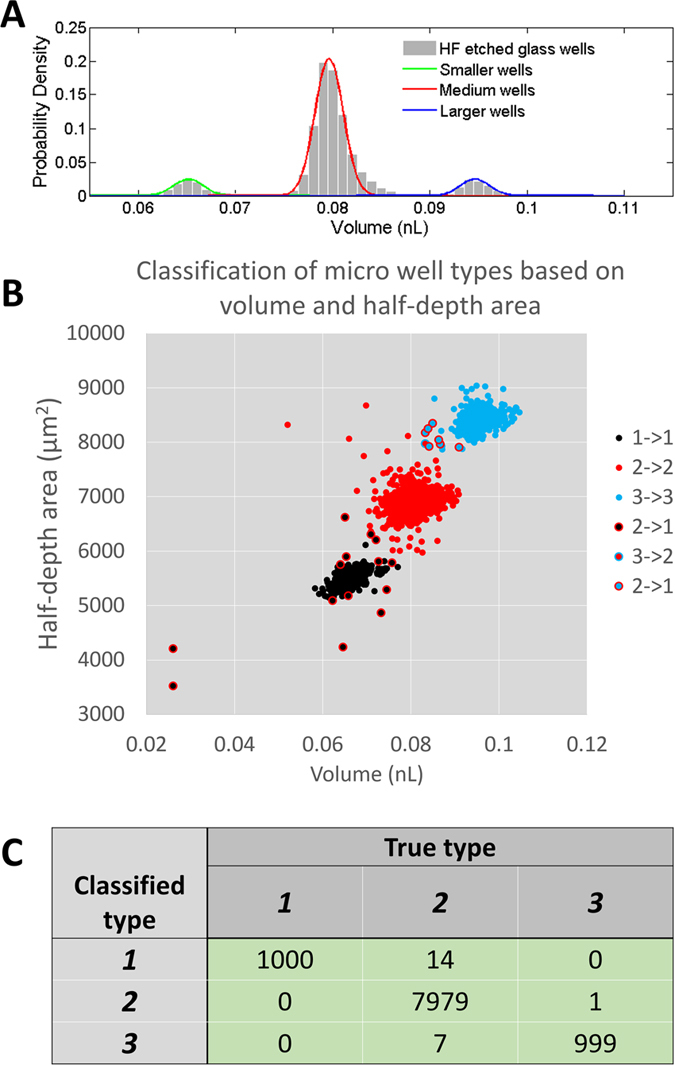
The volume distribution of micro wells in HF-etched glass and laser-ablated PDMS. Samples of 3D profiles of micro wells in glass (**A**) and PDMS (**B**) obtained in single scan, 4 scans and 9 scans, from top to the bottom. The volume distribution of the glass and PDMS micro wells is shown in (**C**,**D**), respectively.

To demonstrate the detection of systematic volume differences in a well array we designed and fabricated a 10,000-well array with 3 different types of wells: (1) 1000 wells with a theoretical diameter of d = 70 μm; (2) 8000 wells with a theoretical diameter of d = 80 μm; and (3) 1000 wells with a theoretical diameter of d = 90 μm. The volumes associated with the 10,000 array wells appear as three well-resolved distributions at 0.065, 0.08 and 0.095 nL, respectively (Fig. 7A). In addition, we plotted a volume versus half-depth-area scatterplot in which three distinct groups are obvious. In order to ‘type’ each well, we used a K-means clustering algorithm commonly used in data mining to assign each well to each group. The spots in the scatter plot were classified into three groups, each corresponding to one of the types of wells we designed (Fig. 7B). The accuracy of the classification is shown in Fig. 7C with only 22 misclassified wells out of a total of 10,000 wells. This result demonstrated that the scanner-acquired full-size 3D profiles can be used to detect systematic differences of sub-groups in a micro-well array, and accurately categorized the well types.

## Conclusions

We have established a scanner-based profilometry method for the examination of micro-structures. The reported method is based only on a two-hundred dollar office scanner and features relatively large inspection area at level of 2000 mm^2^ with ~2 μm resolution at maximum. Using this method, we demonstrated (1) the inspection of the longitudinal cross-sectional variation of laser-ablated PDMS channels; (2) determination of the volume in ‘sagged’ PeT chambers following lamination as well as the corrected volumes provided by improved fabrication methods; (3) the examination of micro well arrays volume distribution fabricated by different methods as well as the multiple subgroups of micro wells in a single array. Some of these inspection tasks have revealed unprecedented quality assessment either in whole-population scale (micro well array) or in large inspection area (PeT chambers). The applicability of the method has been demonstrated at different depth ranges, from hundreds of micrometers (PeT chambers) to tens of micrometers (HF-etched glass and laser-ablated PDMS channels) and even with shallower structures having depths equal to or less than 10 micrometers (micro-wells). The flatbed scanner based profilometry has been established as a low-cost, user-friendly and high-inspection-FOV method for the quality assessment of various types of transparent, enclosed microstructures.

## Additional Information

**How to cite this article**: Xu, K. *et al.* The µSCAPE System: 3-Dimensional Profiling of Microfluidic Architectural Features Using a Flatbed Scanner. *Sci. Rep.* **6**, 22246; doi: 10.1038/srep22246 (2016).

## Supplementary Material

Supplementary Information

## Figures and Tables

**Table 1:** Volume distribution of glass micro wells with increasing HF etching depth.

HF etched depth (μm)	6.8	10.9	14.4
Mean (nL)	0.0482	0.0880	0.1184
Variance (nL)	0.0011	0.0013	0.0014
Coefficient of variation	2.5%	1.5%	1.2%

**Table 2:** Volume distribution of PDMS micro wells with increasing laser power.

Laser power (%)	0.5	1.0	1.5
Mean (nL)	0.0226	0.0454	0.0684
Variance (nL)	0.0023	0.0041	0.0048
Coefficient of variation	10%	9%	7%
