# Supplementary material for: The μSCAPE System: 3-Dimensional Profiling of Microfluidic Architectural Features Using a Flatbed Scanner
Source: Sci Rep. 2016 Feb 29;6:22246. doi: 10.1038/srep22246 (PMC4770298; doi:10.1038/srep22246)
Supplement: Supplementary Information [file srep22246-s1.pdf]

For Submission to Scientific Reports

Supplementary Information to

The µSCAPE System: 3-Dimensional Profiling of Microfluidic Architectural Features Using a Flatbed Scanner

Kerui Xu[1], Qian Liu[1], Kimberly R. Jackson[1], James P. Landers[1,2,3]

Departments of [1]Chemistry, [2]Mechanical and Aerospace Engineering, and [3]Pathology,

University of Virginia, Charlottesville, VA 22904, USA

1. Scanning mode selection

"Reflective" is the most commonly used scanning mode for the scanning of documents, photos and majority of the previously reported analytical applications, in which the light source and the detector are on the same side of the object. However in this mode, the detected incident light in the absorbance calculation is the reflected light from the white pad above the object, which may not have a uniform reflection rate due to the imperfectness of the pad surface. In addition because the detector and the light source are on the same side there is a position shift between them, so if the object has significant thickness there is a problem of shadow. While in "film scan" mode, the detector and the light source are on the different sides of the object, which ensures that the incident light is directly from the uniform light source, and no shadow will be detected since the beam is right below the detector (Figure S1).

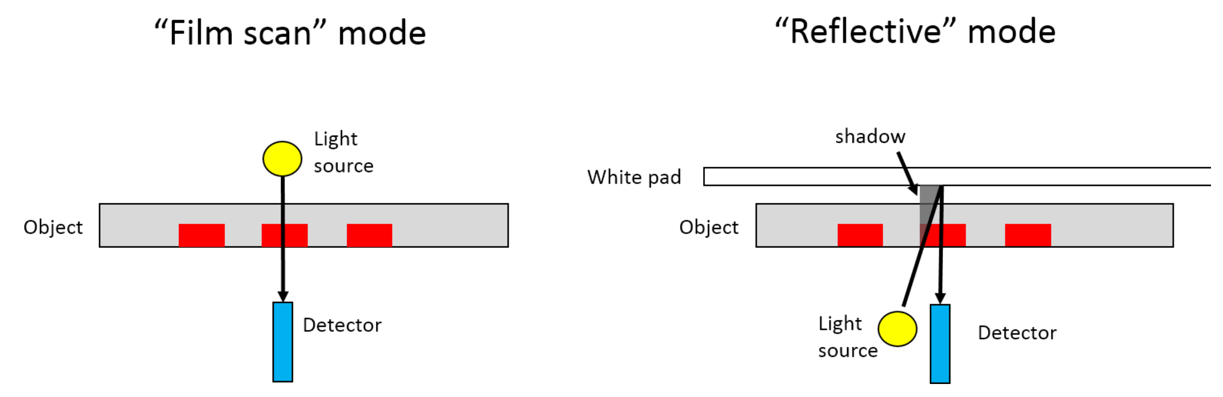

Figure S1. The light-detector setting of different scanning modes

2. Solvent selection

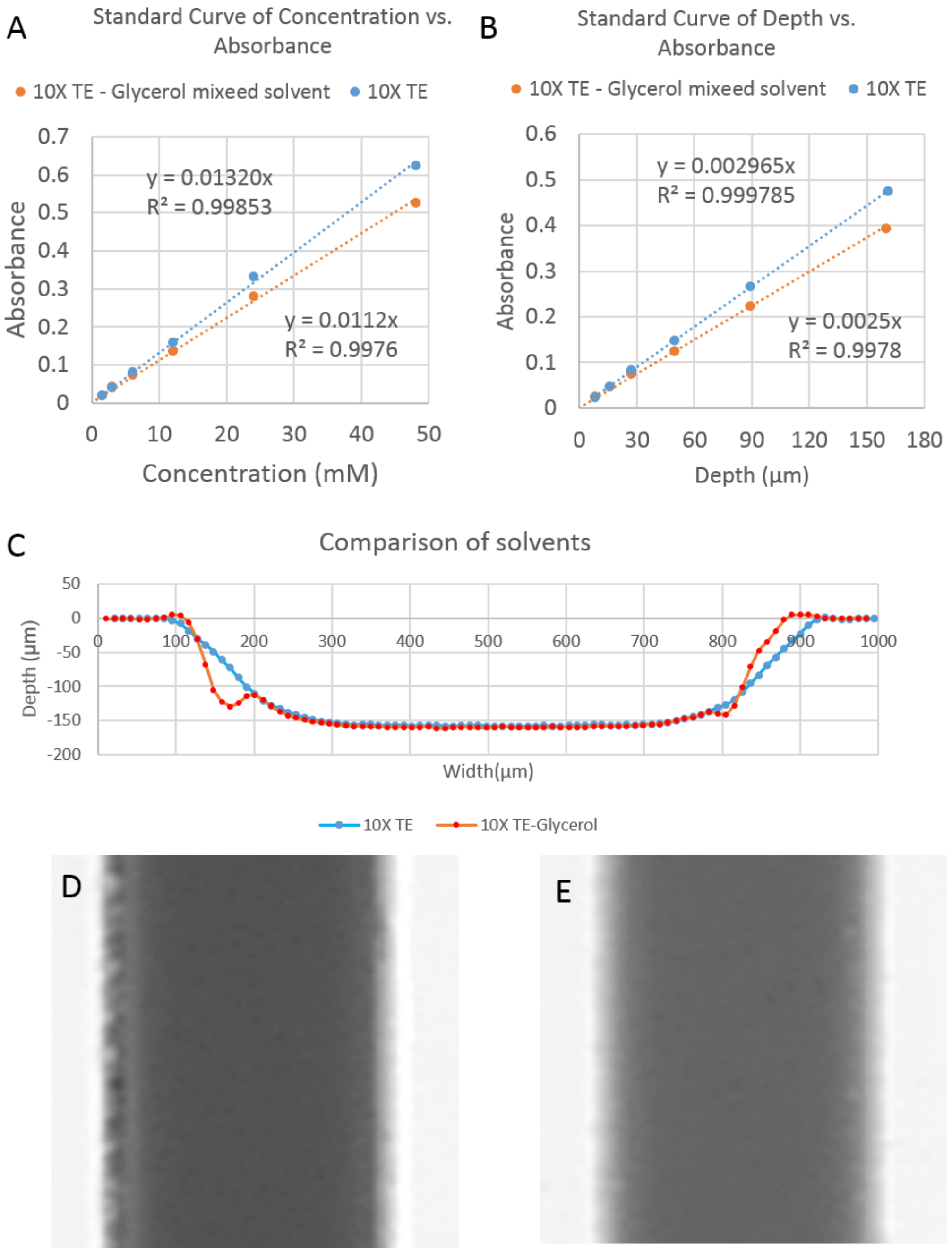

Figure S2. The effect of different solvent on the scanned image. (A) The calibration curve of absorbance vs. concentration in either 10X TE and mixed solvent. (B) The calibration curve of absorbance vs. depth in either 10X TE and mixed solvent. (C) The comparison of the profile of the HF-etched glass channel's cross-section in either 10X TE and mixed solvent. (D-E) The scanned image of glass channel filled with either 10X TE (D) or mixed solvent (E).

In the selection of the solvent for the dye solution, both water and glycerol-water mixture are tested. The calibration curve obtained in both solvent shows good linearity and the allura red in 10X TE has slightly larger coefficient of extinction (Figure S2,A&B). However, the profile of the channels filled with solvent of non-matching RI (water) or matching RI (glycerol-water mixture) show significant differences (Figure S2, C). The bottom part of the channel is quite similar between these two solvents, but at the channel's wall the profile of the aqueous solvent-filled channels presents abnormal bright and dark strips, while the glycerol-water mixed solvent gives a normal cross-sectional profile (Figure S2, D & E). The reason for the distorted profile using water solvent might be the reflection of the light at the interface of aqueous solvent and glass due to the unmatched refractive index. In addition, the glycerol-water solvent provides another advantage that it has a much slower evaporation rate, which is more desired in keeping a consistent concentration of the dye solution during scanning. So we used glycerol-water mixed solvent for all the following profiling tests.

3. Theoretical relation between sagging volume and chamber radius

Assuming the one-dimensional expansion of the polyester film under heating is anisotropic with a constant ratio α, supposing two chambers with radius r1 and r2, then the ratio of the these two chamber's new cap areas after the expansion, A_1/A_2 is:

$$\frac{A_1}{A_2} = \frac{2\pi(r_1)^2(1+\alpha)^2}{2\pi(r_2)^2(1+\alpha)^2} = \frac{(r_1)^2}{(r_2)^2} \qquad (1)$$

Assuming the shape of the sagged surface is a spherical cap, then the surface ratio could also be expressed as:

$$\frac{A_1}{A_2} = \frac{2\pi h_1 r_1}{2\pi h_2 r_2} = \frac{h_1 r_1}{h_2 r_2} \qquad (2)$$

Where h1 and h2 are the height of the spherical-cap shaped sagged volume of chambers.

Combining (1) and (2),

$$\frac{r_1}{r_2} = \frac{h_1}{h_2} \qquad (3)$$

The ratio of the sagging volumes (i.e. the volumes of the concave spherical caps) of these two chambers is:

$$\frac{V_1}{V_2} = \frac{\frac{\pi}{6} h_1 (3{r_1}^2 + {h_1}^2)}{\frac{\pi}{6} h_2 (3{r_2}^2 + {h_2}^2)} \qquad (4)$$

After proper transformation giving eq.(3), eq. (4) becomes:

$$\frac{V_1}{V_2} = \frac{{r_1}^3}{{r_2}^3} \qquad (5)$$

Which shows that the decreased volume is proportional to r^3. However for a non-sagging cylinder chamber with fixed height the designed volume is only proportional to r^2. This explains the trend that the deviation of the volume from the designed volume becomes larger and larger as the radius of the chamber increases, and the reduced volume is proportional to the cubic of chamber radius shown in Figure 4C in the main text.

4. Distribution of the volumes of micro wells by different fabrication conditions

The distributions of the different volumes of micro wells achieved by either HF etching or laser ablation is shown in Figure S3.

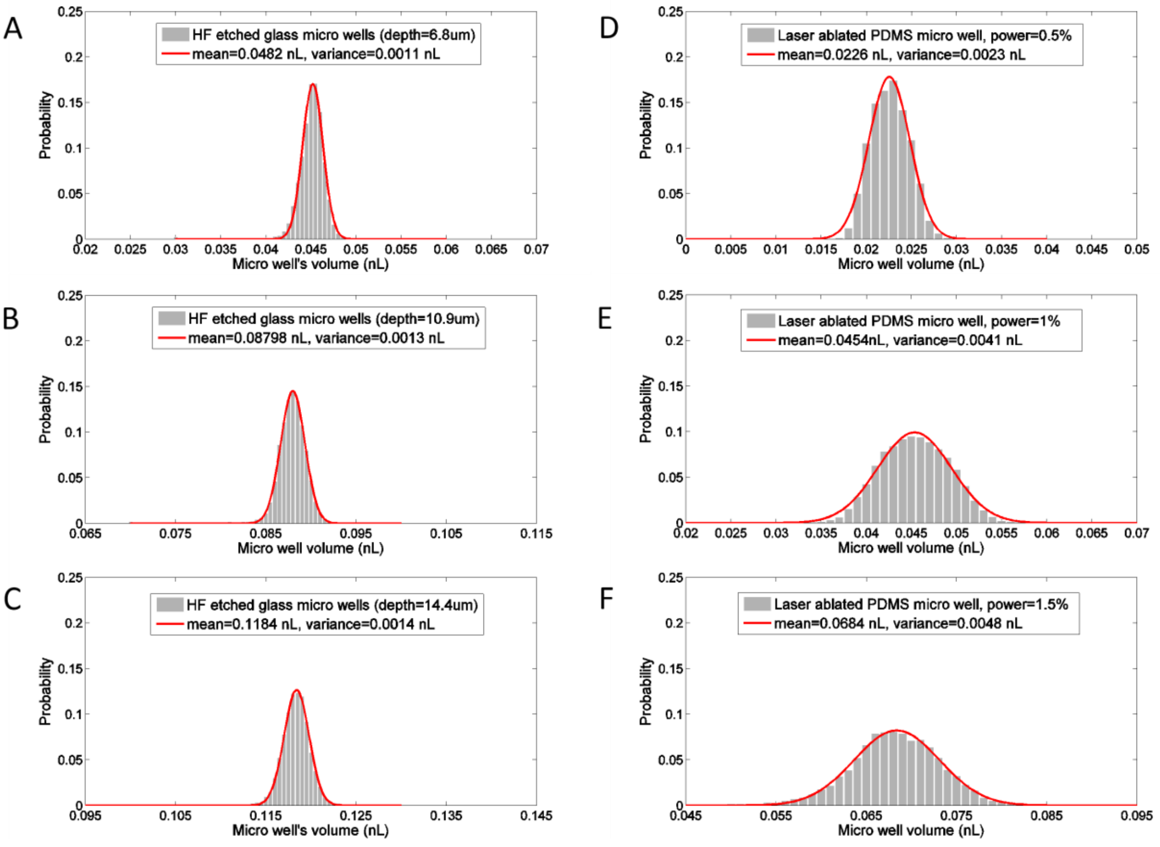

Figure S3. The distribution of different volumes controlled by fabrication processes. (A-C) glass micro wells with increasing HF etching time, giving depths of 6.8, 10.9 and 14.4 μm, respectively. Depths are measured by stylus profilometry. (D-F) PDMS micro wells with increasing laser power at 0.5, 1 and 1.5% respectively.

Because the geometries of the HF-etched glass wells are regular the theoretical volume of the micro-well can be calculated.

The ideal cross-section of a micro-well across the diameter, assuming perfect isotropic etching, has equal lateral etching and vertical etching distance (a=b), as shown in Figure S4 (left). However, the real etching has faster vertical etching rate than vertical etching rate (a>b)[1], as shown in Figure S4 (right).

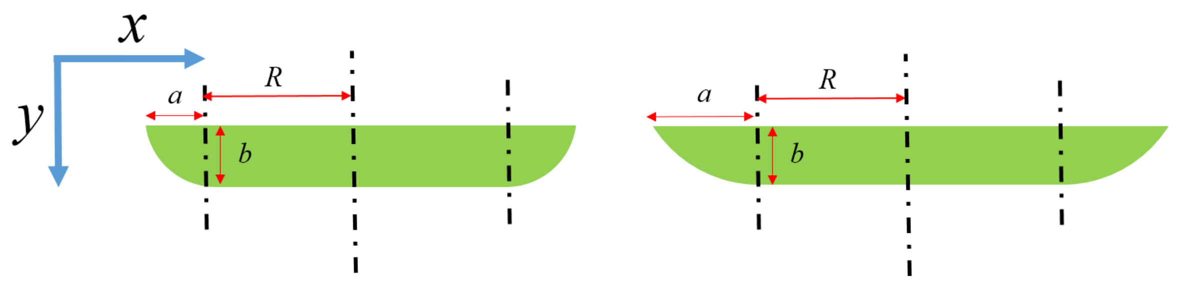

Figure S4. The ideal cross-section (left) and real cross-section (right) of HF-etched glass micro-well.

The volume of the micro-well can be calculated as follow:

$$\int_0^b \pi\left(R+\sqrt{a^2(1-\frac{y^2}{b^2}}\right)^2 dy$$

where *a,b and R* are the lateral-etching distance, vertical-etching distance and the initial radius of the opened window on the mask. After proper change of variable ($\sin\theta = \frac{y}{b}$), the integral is converted to:

$$\int_0^{\frac{\pi}{2}} \pi\left(R+\sqrt{a^2(1-(\sin\theta)^2}\right)^2 \cos\theta\, d\theta = \int_0^{\frac{\pi}{2}} (R+a\cos\theta)^2 \cos\theta\, d\theta$$

The result of the integral is:

$$\pi b(R^2 + \frac{2}{3}a^2 + \frac{1}{2}a\pi R)$$

The lateral etching distance *a* was reported to be 27% longer than the vertical etching distance *b*[1] so *a* can be replaced by 1.27*b*. After plugging the etching depths (6.8, 10.9 and 14.4 µm) and initial radius of the mask window *R*=40µm, the theoretical volumes of the micro-wells are calculated and compared with the µSCAPE-revealed means of the volume distribution in Figure 5S.

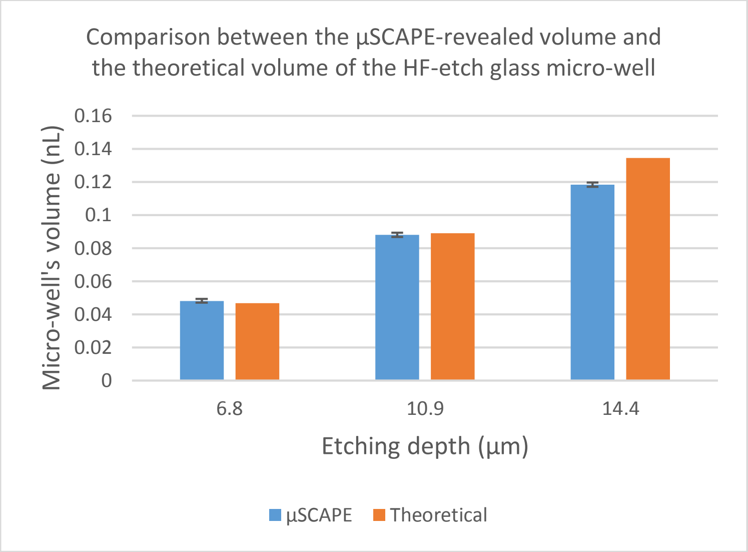

Figure S5. The Comparison between the µSCAPE-revealed volume and the theoretical volume of the HF-etch glass micro-well. Error bar shows the magnitude of the variance of the volume distribution

REFERENCE

[1] M. Bu, T. Melvin, G. J. Ensell, J. S. Wilkinson, and A. G. R. Evans, "A new masking technology for deep glass etching and its microfluidic application," *Sensors Actuators, A Phys.*, vol. 115, pp. 476–482, 2004.
